# The Scent of a Therapy for Spinal Cord Injury: Growth Factors and Their Potential to Modulate Olfactory Ensheathing Cells

**DOI:** 10.3390/biom16010086

**Published:** 2026-01-05

**Authors:** Tobias S. G. Seeberger, Mariyam Murtaza, Andrew J. Rayfield, James A. St John, Ronak Reshamwala

**Affiliations:** Clem Jones Centre for Neurobiology and Stem Cell Research, Institute for Biomedicine and Glycomics, Griffith University, Parklands Drive, Gold Coast, QLD 4222, Australia; tobias.seeberger@griffithuni.edu.au (T.S.G.S.); m.murtaza@griffith.edu.au (M.M.); a.rayfield@griffith.edu.au (A.J.R.); j.stjohn@griffith.edu.au (J.A.S.J.)

**Keywords:** olfactory glia, cell transplantation, nerve repair, combination therapy, cell modulation

## Abstract

Spinal cord injury (SCI) is a debilitating condition resulting in a range of neurological impairments up to complete loss of function below the level of injury. With current clinical management limited to decompression and stabilisation of the injury, there is urgent need to develop effective restorative treatments. In animal models, cell transplantation therapies are being tested that utilise different cell types including olfactory ensheathing cells (OECs), a type of glial cell, to support and promote regeneration. While OECs have a unique combination of properties highly suitable for SCI repair, their efficacy and consistency need to be improved. Evidence suggests a combinational approach using growth factors or compounds alongside OECs may stimulate their innate properties and alter the internal milieu of an injury site in favour of neural repair. Naturally, there is intricate interplay between various growth factors and OECs during development of the olfactory system, and in injury and repair events, which regulate their migration, phagocytosis, and proliferation. Therefore, exploiting different growth factors to selectively enhance OECs’ therapeutic potential could lead to restorative treatment of SCI. While some studies have already explored using growth factors to treat SCI in animal models, an optimal ‘cocktail’ has yet to be identified. In seeking to identify such a cocktail, this review presents the current understanding of SCI and the therapeutic potential of OECs and explores combined use of growth factors and OECs to improve treatment outcomes.

## 1. Introduction

Spinal cord injury (SCI) is a debilitating, life-altering injury that commonly results from severe traumatic events. Depending on the extent of an injury, SCI leads to varying degrees of functional deficits including loss of motor, sensory, and/or autonomic function [1,2]. Current clinical treatments for SCI are limited to decompression and stabilisation of the injury site followed by acute and subacute in-patient rehabilitation, and management of complications [3,4,5,6]. The devastating impact of SCI is therefore compounded by a lack of restorative therapies that can repair damaged tissues and promote neural regeneration.

**Figure 1:**
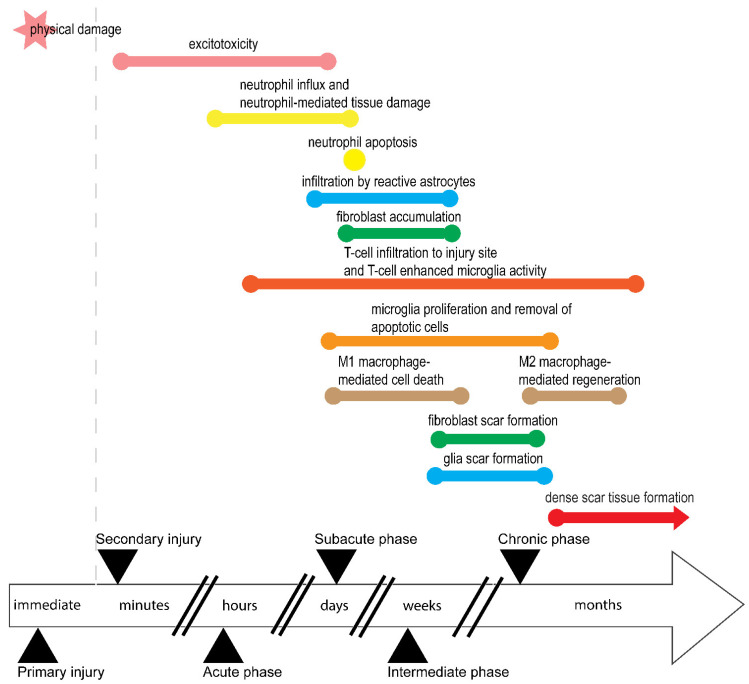
Timeline of spinal cord injury. Events leading to spinal cord injury and subsequent poor regeneration. Primary injury phase is the initiating injury for SCI that leads to secondary injury, which further damages spinal cord and surrounding tissue. The secondary injury phase gives rise to acute, subacute, and intermediate phases, and eventually, a chronic phase during which the site shows scar tissue preventing cells from entering the lesion area. The precise time durations of these phases may vary depending on species and injury type.

In addition to largely irreversible loss of bodily functions, SCI has several unique features that often further complicate the injury, its clinical management, and its impact. SCI has two components: primary and secondary injury (Figure 1). A primary injury is the result of physical impact on the spinal cord and often determines injury severity [7]. This is followed by a secondary injury phase that results from the body’s response to primary injury and leads to further neurodegeneration that effectively expands or exacerbates the primary injury [8]. This secondary injury phase involves excitotoxity, inflammation, and apoptosis of neuronal and supporting tissue. In rats, this continues for approximately two weeks after injury [9]. These key events of the secondary injury phase are discussed in detail below.

SCI also involves a cascade of pathological processes, beginning with excitotoxicity, which occurs within minutes of injury. In excitotoxicity, excessive glutamate is released, damaging oligodendrocytes and leading to demyelination and neuronal death, thereby expanding the injury and worsening functional loss [10,11,12,13,14]. Glutamate is likely released by damaged or dying neurons [15,16,17] of the spinal cord following injury.

Inflammation and immunocytes during secondary injury then begin breakdown of the blood–spinal cord barrier, allowing immune cells to infiltrate the spinal cord [18]. Neutrophils are first responders, arriving within hours and peaking by day one post-injury in most rat SCI models [10,19,20]. However, neutrophil peaking at the SCI site is also SCI-type dependent and species dependent [10]. These neutrophils contribute to tissue damage, neuroinflammation, and scar formation before undergoing apoptosis and disappearing by day ten [21,22].

Macrophages then follow, first infiltrating the site between initial hours [23] and three days post-injury [20]. These cells, derived from blood monocytes, play dual roles depending on their phenotype. M1 macrophages, induced by pro-inflammatory cytokines, exacerbate inflammation and cell death, while M2 macrophages, stimulated by anti-inflammatory cytokines, support tissue repair and regeneration [24,25,26,27,28]. M2 macrophages are also shown to influence scar formation by stimulating astrocytes through transforming growth factor beta (TGF-β) secretion in vitro [29,30,31]. Macrophages infiltrate in two waves: an early M1-dominated wave peaking at day seven, and a later M2 wave beginning around day 14 and persisting for months [32,33,34]. Despite prolonged presence of M2 macrophages that participate in repair processes and anti-inflammatory responses, injury environments tend to favour M1 dominance. This suggests that M1 and M2 macrophages perform multiple roles during secondary injury phases, ranging from clearing the injury site to participating in repair processes and potentially glial scar formation, thus exhibiting high plasticity and overlapping properties between subtypes [35].

The resident immune cells of the CNS, microglia, also exhibit M1 and M2 phenotypes [36,37]. They begin proliferating around 48 h post-injury, peak at day seven, and remain active for at least 42 days post-SCI [38], contributing to inflammation and chronic injury, and likely participating in removal of apoptotic debris during secondary injury phase. In vivo studies, however, are yet to confirm whether and how M2 microglia also polarise reactive astrocytes to form the glial scar.

T-cells infiltrate the injury site about 12 h post-injury, also peaking at day seven and persisting for up to 180 days in rats [19,32]. These adaptive immune cells promote inflammation and enhance microglial activity, contributing to chronic inflammation and tissue degradation [39,40,41,42]. While studies cited above use the classical M1/M2 microglia model, this classical M1/M2 microglia model has seen a shift towards a more dynamic microglia model with different phenotypes [43].

Overall, the secondary injury phase of SCI is marked by a complex interplay of excitotoxicity, immune cell infiltration, and inflammatory responses. While some immune cells contribute to repair, others exacerbate damage, highlighting the dual nature of immune response in SCI pathology.

*Scar* *Formation: Healing or Hindrance?*

Besides immune responses preventing spinal cord regeneration, another concern is formation of scar tissue. While scarring is a natural response of the body to injury, scarring at an SCI site is especially complicated and involves different cell types. SCI induces astrocyte-specific hypertrophic cell body changes in grey and white matter [44]. These astrocytes become reactive, with distinct hypertrophic bodies, and infiltrate surrounding tissue of the lesion within 24 h. They relocate at the central core of the lesion and elongate to find and communicate with neighbouring reactive astrocytes. Through this interaction, reactive astrocytes interlace with each other and form a dense, continuous boundary separating the central core from the surrounding lesion within two weeks post-SCI in rats [44,45,46,47]. Fibroblasts form another layer of scar tissue during SCI, accumulating at the injury site three days post-SCI and forming the fibrotic component of scar tissue with a dense extracellular matrix by day seven post-SCI in rats [48]. This indicates reactive astrocytes and fibroblasts form separate scar tissues that both impede migration of regrowing axons. The composite scar tissue also traps inflammatory cells inside the SCI lesion core to protect surrounding undamaged spinal cord tissue instead of promoting neuronal regeneration [44,47].

Thus, immunocytes, inflammatory reaction, scar tissue, and debris of injured and damaged native cells together make a hostile injury site which is non-conducive to regrowth, repair, or regeneration. As a result, most cases of SCI cause permanent loss of neurological function, drastically reducing quality of life for the individual.

*Cell- and Biomaterial-Based Experimental Therapeutic Approaches*

Several experimental biological therapeutic approaches are under exploration to achieve tissue repair and regeneration, and to return lost functions. These include using cell transplantation, biomolecules, or biomaterials [49]. For example, cell transplantation approaches to treat SCI include stem cells such as neural stem cells, mesenchymal stem cells [50,51] or oligodendrocyte precursor cells [52,53], and differentiated cells such as Schwann cells [54] and olfactory ensheathing cells (OECs) [55,56]. Other approaches utilise biomolecules such as growth factors to promote repair [57,58]. Biomaterial-based approaches to treat SCI include hydrogels or scaffolds that induce repair by providing nerve guidance and mechanical stability [59,60,61].

Olfactory ensheathing cells (OECs), glial cells of the primary olfactory system, are a particularly promising candidate for cell transplantation therapy approaches. A concluded phase I clinical trial using purified autologous OECs has shown their transplantation to be safe for human patients [62], and another concluded phase I clinical trial has demonstrated efficacy to improve motor and sensory function in at least one human patient with chronic SCI (NCT Number: NCT01231893) [63]. An additional trial showed OEC transplantation into an SCI site is feasible, safe, and did not cause adverse effects [62]. In this case, although two out of three treated patients did not show functional changes, one treated patient reported sensory improvements [62]. Another clinical trial that combined OEC transplantation with pre- and postoperative neurorehabilitation showed a range of improvements on the ASIA scale for participants, with some improved motor and sensory function [63]. There have been several more clinical trials over the past 20 years showing the use of OECs to be safe; however, they exhibit varying levels of efficacy. This variability and unpredictability of outcomes is the most likely obstruction to further clinical testing and implementation of OEC-based therapy. Therefore, therapeutic use of OECs needs further refining [49].

Use of OECs comes with some challenges, including lack of robust identification and purification techniques, unstable surgical transplantation techniques, and poor cell survival following transplantation [56,64]. A better understanding of OECs’ natural abilities and functions, especially in the context of reparative neurobiology, is crucial to overcome these barriers. With this understanding, several studies have tried to stimulate the cells to enhance treatment outcomes using natural compounds [65,66], biomaterials [67,68], (repurposed) pharmaceutical agents [69] as well as growth factors [70,71]. As such, the rest of this review will focus on the therapeutic properties and potential of OECs, with specific focus on growth factors involved in their natural biology, to explore how growth factors could be used to enhance OEC transplantation outcomes for treating SCI.

## 2. Therapeutic Potential of OECs

The mammalian olfactory system has a unique ability to undergo constant and consistent regeneration. Understanding how this functions is crucial in understanding the therapeutic properties of OECs. Dendrites of primary sensory olfactory neurons extend into the nasal cavity, detecting odours while also being exposed to the environment [72,73]. This direct environmental exposure subjects dendrites to microorganisms and toxic chemicals, which results in loss of an estimated 1–3% of sensory olfactory neurons each day [74,75,76,77,78]. The continual loss of primary olfactory neurons is compensated by adult stem cells that line the basal layer of the epithelium and give rise to new neurons [79,80]. These new neurons extend their axons up into the cranial cavity where they target their appropriate odorant receptor-specific glomerulus within the olfactory bulb. This continual regeneration of the primary olfactory nerve is due in part to OECs.

**Figure 2:**
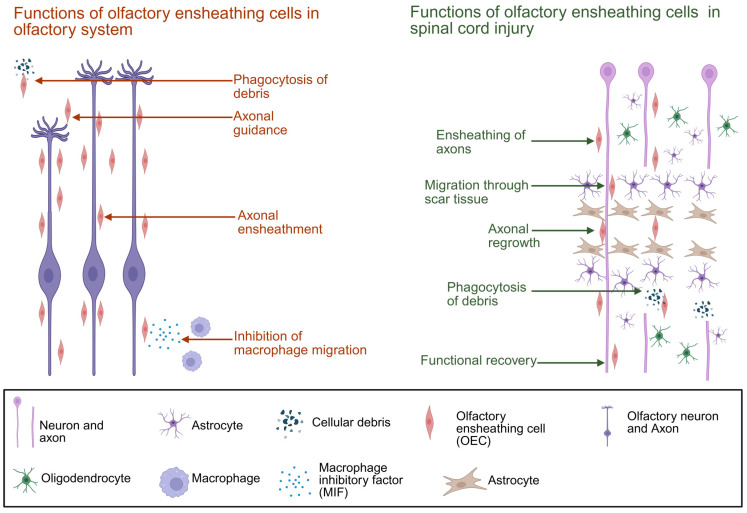
Summary of OEC functions in the olfactory system and after cell transplantation. Key functions of OECs in olfactory system and after transplantation into spinal cord injury site. Created in BioRender. Seeberger, T. (2025) https://BioRender.com/f34j145 (accessed on 15 November 2025).

OECs reside in the lamina propria and outer layer of the olfactory bulb, fulfilling several different functions in the olfactory system. During development and after major injury, OECs lead axons by migrating ahead of them [81,82] and ensheathe fascicles of the olfactory axons [55,81,83]. OECs also express a range of growth factors beneficial for axonal survival [84], and are the primary phagocytes of the olfactory nerve, taking up debris of the damaged axons constantly being replaced [85,86]. They are also suggested to inhibit influx of systemic macrophages by secreting macrophage migration inhibitory factor (MIF) [87]. Interestingly, when co-cultured with other cells, OECs also grow between astrocytes [88], fibroblasts, and neurons from the peripheral and central nervous system in addition to the olfactory neurons from their natural habitat [89,90,91,92]. These numerous mechanisms of action make OECs an attractive option for cell transplantation to treat SCI (Figure 2); however, some challenges remain.

OECs can be obtained by biopsy of the olfactory mucosa or olfactory bulb. While sourcing OECs from the olfactory bulb may yield higher purity cultures than mucosal biopsies [64], extraction of bulb OECs requires a craniotomy in an operating room [93] and potential permanent loss of olfactory function. Olfactory bulbs also contain neural progenitor cells in addition to OECs, which contributes to the differences between mucosal and bulb-derived OECs’ cellular composition [94,95,96]. In contrast, mucosal OEC extraction can be performed through intranasal endoscopy in an outpatient setting [97] and does not lead to loss of sensitivity of smell function.

While a meta-analysis shows that OEC transplantation to treat SCI can lead to functional recovery [98], reviews have identified that few OECs survive in the injury site long term [56,99]. This may be the result of inflammatory cells such as neutrophils and macrophages and the presence of pro-inflammatory cytokines [20]. Evidence also shows that OECs can be classified into several subtypes (mucosal and bulb-derived OECs, for example) [100,101], meaning that neurotherapeutic potential may differ between OEC subpopulations. Therapeutic effects of transplanted OECs may also be temporally limited, improving motor function during the first months post-transplantation, but having less impact over time [102]. Thus, clinical application of OECs faces certain challenges as they require enhancement of innate properties to improve integration and therapeutic efficacy in an SCI environment. This leads to use of growth factors alongside OECs as a potential solution, providing additional support for OECs and improving their activity and survivability in SCI treatment.

## 3. Growth Factors Stimulating OECs

Growth factors are small molecules secreted by cells to trigger and modulate behaviours such as differentiation, growth, proliferation, and repair and regeneration. Growth factors also play key roles in OEC activity during development, physiological functions, and regeneration of the olfactory system [103,104,105].

### 3.1. Growth Factors Involved in Development of Olfactory System

Growth factors mediate different functions in OECs such as proliferation [106] and morphological changes [107]. OECs also secrete neurotrophic growth factors such as brain-derived neurotrophic factor (BDNF), nerve growth factor (NGF), and glia-derived nerve growth factor (GDNF) [84,108]. Through these different growth factors, OECs function to guide axons in vitro [109,110,111].

Additionally, in vitro OECs express several receptors for growth factors during development, such as low-affinity nerve growth factor receptor, GDNF family receptor alpha-1, tyrosine kinase receptor, and p75 nerve growth factor receptor [112,113,114,115], and show differences in expression depending on location and developmental stage. Spatiotemporal analysis of embryonic mouse heads at E11.5 to E15.5 showed that OECs also migrate ahead of neurons within the developing olfactory system to create a microenvironment beneficial for neuronal migration in vivo [114,116]. This indicates growth factors act on OECs differently at various stages of development. While there are numerous growth factors that may interact with OECs, we will now focus on a select few that have been reported to potentially stimulate OECs.

Platelet-derived growth factor (PDGF): PDGF is secreted by platelets from damaged blood vessels, acting on the vessels and surrounding mesenchymal tissue to mediate blood vessel repair. In the CNS, PDGF acts on perivascular astrocytes via PDGF-A receptors to increase blood–brain barrier permeability [117]. PDGF is also expressed in human brain tissue and shows neuroprotective properties following ischemic stroke by improving vascularisation [118,119]. Schwann cells also express PDGF-B receptors, which can increase cell proliferation upon activation in vivo [120]. Despite glial cells such as astrocytes and Schwann cells expressing PDGF receptors, some evidence suggests that the PDGF receptor may be only transiently expressed in the olfactory system during its development in utero and in the early neonatal stage [121,122,123]. PDGF-B receptor activity has been implicated in the regeneration of olfactory neurons in vivo [121], but adult OECs may not express PDGF receptors in vitro [124]. This raises an interesting possibility that they may differentially express PDGF receptors in vivo versus in vitro, given that OECs apparently respond to PDGF signalling in vitro.

Despite previous research suggesting OECs express PDGF receptors transiently in vivo, receptor presence might be conditionally dependant on environmental cues. Therefore, PDGF treatment of OECs may prove to be an effective approach to induce proliferation prior to or after transplantation into an SCI lesion site, possibly increasing their reactivity and proliferative potential.

Glia-derived neurotrophic factor (GDNF): GDNF is secreted by glial cells and can induce morphological and functional changes in OECs by binding to a RET1 and GFRα-1 receptor complex [112]. This activation of the RET1/GFRα-1 receptor complex then promotes in vitro migration of OECs, including those which may be governed by c-Jun N-terminal kinase and Src protein kinase activity [112]. Furthermore, GDNF also directly induces morphological changes in OECs in vitro, decreasing somatic size, increasing length of processes, and increasing actin and tubulin expression [112]. Other in vitro studies show that GDNF is essential for OECs to form extrusions such as peripheral lamellipodial extrusions, which are involved in cell–cell interaction and OEC migration [125]. Lamellipodial wave activity in retrograde or anterograde direction is essential for regulating cell–cell adhesion and migration of OECs and is regulated by GNDF but independent of the leading edge in vitro [126]. In in vitro models of the spinal cord specifically, GDNF has been shown to promote OEC migration through white matter [112].

Therefore, GDNF treatment is a potential approach for increasing OEC migration, by increasing process length, decreasing cell size, and upregulating lamellipodia wave activity. This combination of events is likely to increase OEC migration through white matter of injured spinal cord [112], a trait crucial for their therapeutic potential.

Semaphorin 3A: Semaphorin 3A receptors neuropilin-1 and neuropilin-2 are expressed on OECs during development of the olfactory system in vitro [115]. Experiments have shown that a semaphorin 3A gradient prevents OEC migration in vitro, resulting in collapse of the OEC leading front [115]. Thus, expression of neuropilin-1 and neuropilin-2 receptors in vitro may result in inhibition of OEC migration, contributing to formation of the olfactory nerve layer during olfactory system development in rats [115]. In this regard, semaphorin 3A could help regulate OEC migratory ability in a neuro-reparative context.

### 3.2. Growth Factors Involved in Regeneration of Olfactory System

Brain-derived neurotrophic factor (BDNF): The natural role of BDNF lies along neuronal developmental pathways, including synaptic plasticity and cognition. Interestingly, dysregulation of BDNF is implicated in Alzheimer’s disease and other neurodegenerative disorders. In the olfactory system, BDNF is involved with natural regeneration. BDNF expression increases during regeneration of the olfactory epithelium and olfactory bulb, potentially leading to BDNF-induced cell renewal in vivo [127]. This indicates BDNF may be suitable to improve SCI treatment outcomes using OECs, although more directed research is warranted to confirm this.

Fibroblast growth factor 2 (FGF2): Another growth factor involved in regeneration of the olfactory system is FGF2, and adult OECs express FGF receptor 1 in the lamina propria below the basement membrane and surrounding olfactory nerve bundles in vitro [128]. Expression of FGF2 increases within 12 h following a chemical injury to the olfactory system in experiments, resulting in upregulated FGF2 that persists for up to three days post-injury [129]. Interestingly, FGF receptor 1 mRNA expression also increases within the first 12 h and remains upregulated for up to 14 days post-injury in vivo [129]. The same study has shown that FGF2 administration enhances regeneration of the olfactory epithelium in vivo [129]. Thus, FGF2 and FGF receptor 1 are upregulated once the olfactory system is injured, implying they are both involved in olfactory system regeneration.

**Figure 3:**
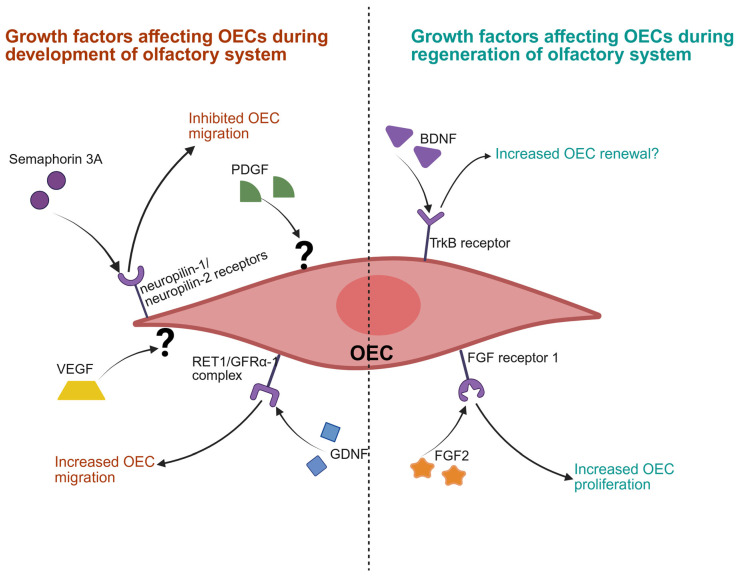
Summary of growth factors affecting OECs during development and regeneration of the olfactory system. Different growth factors affect OECs depending on the state of the olfactory system and developmental stage (BDNF = brain-derived neurotrophic factor; FGF1/2 = fibroblast growth factor 1/2; GDNF = glial cell line-derived neurotrophic factor; OEC = olfactory ensheathing cell; PDGF = platelet-derived growth factor, RET1/GFRα1 = receptor tyrosine kinase1/glial cell line-derived neurotrophic factor receptor alpha 1; TrkB receptor = tropomyosin receptor kinase B); ? = unknown receptor. Created in BioRender. Seeberger, T. (2025) https://BioRender.com/z63w585 (accessed on 15 November 2025).

One recent study used novel bioactive, degradable hydrogel to deliver FGF and interleukin-10 and successfully induced recovery following SCI in rats [130]. An earlier in vitro study also showed that FGF2 has mitogenic and survival effects on olfactory spheroid cultures of neonatal OECs [131]. Yet another study has shown that FGF2 increases proliferation of neonatal OECs within a narrow dosage range [132]. Taken together, these studies suggest that FGF2 and OECs work together during regeneration of the olfactory system. Therefore, exogenous administration of FGF2 combined with OEC transplantation to treat SCI warrants further exploration towards its therapeutic potential (Figure 3).

### 3.3. Application of Growth Factors for Enhancing OECs’ Neurotherapeutic Potential

Several growth factors and their effects on OEC stimulation for transplantation into an SCI lesion site have been explored in vitro as well as in vivo. This segment focuses on growth factors that have been tested for enhancing outcomes of OEC transplantation-based SCI repair. Specifically, BDNF, transforming growth factor beta 1 (TGF β1), FGF 2, PDGF, VEGF, and GDNF are discussed here as they have attracted significant focus and relevancy as potential candidates for SCI treatment alongside OECs (Figure 3).

Brain-derived neurotrophic factor (BDNF): OEC-derived BDNF promotes axonal regeneration of adult retinal neurons in addition to its previously described functions in vitro [71]. Therefore, treatment for nerve injuries involving OECs may have wider applications beyond SCI, including other CNS injuries.

Treatment of sciatic nerve crush injuries using BDNF resulted in increased recovery with the initial response and at 7 and 17 days post-injury, respectively, compared to untreated mice in vivo [133]. This indicates BDNF may prevent atrophy at the distal end of neurons while also promoting neuronal growth following injury. The same study showed exogenous BDNF increases mRNA expression responsible for distal axonal regrowth without affecting endogenous BDNF and GDNF expression, while also upregulating pathways responsible for intrinsic growth capability and delaying demyelination of distal axons at an injury site in vivo [133]. This suggests exogenous BDNF has a direct neuroprotective effect on axons post-injury, supporting its potential for treating SCI.

It has been shown that BDNF released by OECs is necessary for regeneration, although it cannot promote regeneration of adult neurons in vitro on its own [71]. This indicates other growth factors are essential to induce neural regeneration in adults. Specifically, matrix metalloproteinase 2 and BDNF together can induce OEC-mediated adult axon regeneration in vitro [71]. Thus, OEC-derived BDNF works in combination with other factors to promote adult axonal outgrowth in vitro.

The migration of OECs may also be directed using a BDNF gradient. The gradient acts through tropomyosin receptor kinase B and transient receptor potential channel 3, resulting in a transmembrane calcium influx, which promotes OEC migration in vitro [134]. In a rat model of spinal cord hemi-section, however, administration of OEC-expressed BDNF increased pain sensitivity to mechanical and thermal stimuli by regulating extracellular signal-regulated kinase activity [135]. Thus, OECs and BDNF may induce CNS repair, but it would need to be managed to minimise risk of neuropathic pain.

Transforming growth factor beta 1 (TGF-β1): TGF-β1 is an anti-inflammatory growth factor that has a natural role in regulation of cell maturation, differentiation, migration, and apoptosis. OECs release TGF-β1 during phagocytosis of neuronal debris [136] and treating OECs with exogenous TGF-β1 increases both phagocytotic activity of OECs and the presence of phagocytotic OECs [136]. Thus, TGF-β1 is not only expressed by phagocytic OECs, but it also creates a positive feedback loop by which OECs are primed to become more phagocytotic and recruit other OECs to phagocytose apoptotic neurons or their cell debris. Under effects of TGF-β1, OECs in vitro change to a flattened shape that enhances their phagocytotic activity [136], indicating phagocytic OECs undergo morphological changes to be more efficient in the presence of apoptotic neurons or cell debris. On a molecular level, TGF-β1 upregulates the integrin/MFG-E8 signalling pathway in OECs to trigger this enhanced phagocytic activity [136]. While TGF-β1-treated OECs show increased phagocytic activity towards debris of injured neurons, uninjured neurons survive on flattened OECs in vitro [136]. This study highlights that TGF-β1 increases neuronal survival by increasing OEC phagocytotic activity and by changing OEC morphology to be more phagocytotic. These factors positively influence neuronal outgrowth and neuroprotection. Interestingly, endogenous TGF-β1 signalling can improve injury repair and angiogenesis by indirectly stimulating Schwann cell outgrowth in vivo; however, exogenous TGF-β1 does not affect Schwann cell proliferation in a similar manner [137].

TGF-β1 has been shown to induce neurite outgrowth in scratch assays in dopaminergic cells in vitro and lessens pain associated with nerve injury while preventing microglia proliferation in response to SCI in mice [138]. Furthermore, the same study indicates that TGF- β1 prevented astrocytes and microglia activation following SCI while reducing inflammatory responses and reducing expression of monocyte chemoattractant protein-1 (MCP-1), a chemokine responsible for microglia activation [139]. Conversely, TGF-β1 reportedly prevented neurite outgrowth in primary cerebellar granule cells in vitro, while addition of TGF-β1 receptor inhibitor LY364947 restored neurite outgrowth in the cell culture [140]. TGF-β1 was also shown to reduce inflammation and enhance functional repairs after sciatic injury in rats [141]. Similarly, an older study showed that TGF-β1 does not induce motor neuron outgrowth, but does have neuroprotective properties in vitro [142].

With these varying results, the role of TGF-β1 in inducing neurite outgrowth remains unclear. Furthermore, questions remain around efficacy of exogenous TGF-β1 in combination with OECs, and potential therapeutic benefits of OECs pretreated with TGF-β1 or supplemented with TGF-β1 after transplantation in vivo are unknown. Further questions that remain around activity duration of TGF-β1-treated OECs need to be addressed.

Fibroblast growth factor 2 (FGF2): In addition to its regenerative influence on OECs and the primary olfactory system, exogenous administration of FGF2 has been shown to improve nerve regeneration and myelination after peripheral nerve injury in rats, leading to reduction in scar size and reversing nerve atrophy in vivo [143]. FGF2 may be a mitogenic factor for OECs, inducing OECs to undergo mitosis and to myelinate regenerating axons in vitro [107,132]. FGF2 may require temporal and dosing precision, however, since it leads to macrophage accumulation at an injury site, increases engulfment of debris and damaged axons by Schwann cells, and downregulates myelination of regenerated axons [144].

These studies also suggest that FGF2 may increase OEC migration into the periphery of an injury site to clear the area of cell debris and potential pathogens, preventing inflammation and scar tissue formation at and around the injury site post-surgery. While this supports FGF2 as a potential candidate to stimulate OECs to treat SCI in humans, it may excessively increase OEC proliferation and induce a morphological switch to a more migratory phenotype after prolonged application. Whether FGF2 application induces OECs to myelinate regenerating axons and undergo mitosis has also yet to be tested in in vivo studies of SCI.

Platelet-derived growth factor (PDGF): PDGF has two subtypes, PDGF-AB and PDGF-BB, and both subtypes can induce proliferation of bovine smooth muscle cells by inducing DNA synthesis in vitro [145], and combination of the two PDGF subtypes has a synergistic effect on DNA synthesis and mitogenesis in vitro [145]. A recent study has also shown PDGF promotes expression of nerve repair genes and phagocytic properties of OECs in vitro [70]. This suggests PDGF may be suitable for enhancing OEC-mediated therapeutic outcomes of SCI repair. However, whether PDGF can induce cell proliferation in OECs similar to smooth muscle cells remains to be proven. Nevertheless, PDGF is a suitable candidate to potentially modulate OEC activity and improve nerve regeneration when treating SCI in humans as it has been shown to prevent motor neuron loss and induce functional recovery following acute SCI in mice in vivo [57]. However, similar to pre-treating OECs with TGF-β1, the potential and efficiency of OECs pretreated with PDGF or supplementing PDGF after OEC transplantation in vivo remains unknown.

Vascular endothelial growth factor (VEGF): VEGF regulates formation of new blood vessels through angiogenesis in development and select situations, such as wound healing in adults [146]. When applied to OECs, VEGF-treated cells show increased phagocytosis up to 24 h after treatment in vitro [70], suggesting VEGF may be a suitable candidate for an OEC transplantation-growth factor combination approach to SCI treatment. The same study showed that combined treatment of VEGF and PDGF enhances OEC activity, mitigating inflammation and increasing phagocytic activity in vitro [70]. This highlights the potential of VEGF application to OECs, alone or in combination with PDGF, to treat SCI.

In an injured olfactory system, VEGF + PDGF combination therapy has been reported to improve olfactory regeneration after olfactory bulbectomy, increasing the growth of regenerating axons and leading to an increased extension to caudal regions in mice in vivo [147]. This suggests VEGF + PDGF may act on OECs to stimulate regeneration and proliferation of olfactory neurons in vivo. A different study also showed VEGF + PDGF combination treatment reduced macrophage and microglia presence surrounding an SCI lesion, while also increasing the lesion cavity size in rats in vivo [148]. Interestingly, the same study showed VEGF + PDGF combination therapy reduced secondary neurodegeneration following SCI, while the individual treatment using VEGF or PDGF worsened secondary degeneration in vivo [148]. Thus, combination treatments may show a promising approach if potential side effects are accounted for.

Glial cell-derived neurotrophic factor (GDNF): The main function of GDNF includes promoting survival of central and peripheral neurons [149,150]. GDNF treatment as part of treating optical nerve injury using OEC transplantation has been shown to cause flash visual evoked potential one-week post-injury, and restoring more than 90% of baseline evoked potential 8 weeks post-injury in rats in vivo [151]. Interestingly, this therapy also led to increased axon elongation and number of regenerating axons compared to an OEC-only treatment in vivo [151], indicating a synergistic relationship between GDNF and OECs in axonal repair. Although the authors did not test effects of GDNF on OEC morphology and function, the overall results suggest GDNF may be a good candidate for testing with OEC as part of SCI treatment since GDNF may increase OEC activity and mediate axon elongation and regeneration in vivo.

Furthermore, GDNF has been shown to improve functional recovery below the SCI level in rats by reducing secondary injury, because GDNF can cross the barrier between blood and spinal cord [152]. While inducing overexpression of GDNF via lentivirus-mediated *GDNF* gene transfer has been reported to promote motor-sensory recovery after SCI [153], there is a risk of GDNF stimulation causing non-directional growth in axons, leading to axonal tangles or coils rather than functional axonal regeneration [153]. This is where the combination of OECs with GDNF may alleviate risk, as OECs may contribute to axonal guidance. However, the effect of GDNF on OEC function and morphology post-transplantation remains unclear.

### 3.4. Combining Growth Factors and OECs for Treating Spinal Cord Injury

As discussed above, growth factors have a remarkable potential to support and enhance OECs’ natural abilities and therapeutic potential. For their clinical implementation, however, the mode of delivery of growth factors requires thorough consideration. Specifically, while different growth factors help mediate OEC function to guide axonal regrowth and movement, their delivery to OECs during and after transplantation is a key issue. The most important consideration here would be to understand how the different growth factors interact with and regulate SCI and natural injury progression.

Table 1 provides an overview of the growth factors involved with normal physiology of the spinal cord and how their expression levels change post-injury. The table also shows common sources and target cell types for these different growth factors.

Clearly, biological functions considered together with downstream events following on from injury (Figure 1) can highlight injury phases during which growth factors may have an optimal restorative effect. As an example, VEGF and PDGF play a role in neuroprotection and prevention of apoptosis in vivo [57,157,163,164,165,166,167]. Therefore, they would likely have maximum efficacy in early stages of injury, such as the immediate and acute phases. Furthermore, NGF is secreted by the glia to target neurons and help with neuroprotection in vivo [168,171,172,173]. Similarly, GDNF and BDNF tend to enhance axonal outgrowth and synaptic plasticity and support remyelination in vivo [152,154,155,156,166,168,169,170], and thus may have better efficacy in subacute or intermediate injury phases, especially if debris at the injury site has been cleared. NT-3 may be another potential growth factor as it has been shown to promote proliferation and survival in oligodendrocytes [174]. Conversely, FGF2 and TGF-β1 are involved in proliferation of reactive astrocytes and scar formation as well as neurite outgrowth in vivo [157,158,159,160,161,162], implying application of these growth factors would need to be precisely modulated and timed to alter outcomes in a favourable way and reduce risk. When combined with OECs, the effects of each growth factor may also vary.

**Figure 4:**
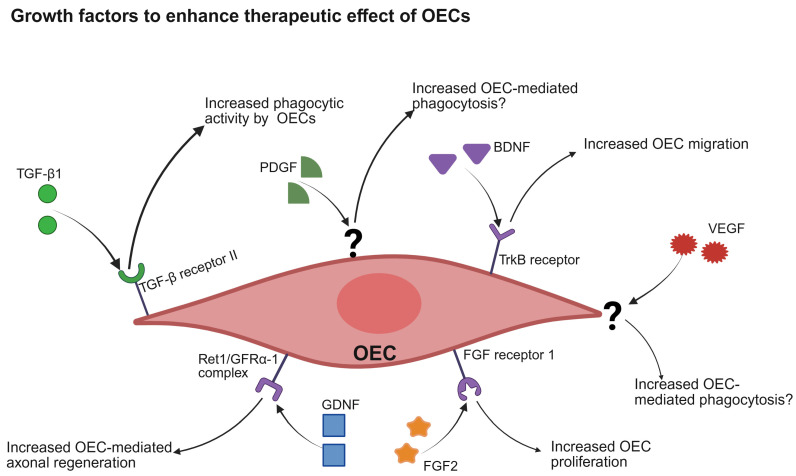
Summary of potential growth factors to stimulate OECs after transplantation. Growth factors induce different OEC activities after cell transplantation, resulting in different, potentially beneficial outcomes (BDNF = brain-derived neurotrophic factor; FGF1/2 = fibroblast growth factor 1/2; GDNF = glial cell line-derived neurotrophic factor; OEC = olfactory ensheathing cell; PDGF = platelet-derived growth factor, RET1/GFRα1 = receptor tyrosine kinase1/glial cell line-derived neurotrophic factor receptor alpha 1; TGF-β1 = transforming growth factor beta 1; TrkB receptor = tropomyosin receptor kinase B; VEGF =vascular endothelial growth factor). ? = unknown receptor. Created in BioRender. Seeberger, T. (2025) https://BioRender.com/b14s466 (accessed on 15 November 2025).

The transient nature of growth factors also remains a challenge to providing fine control over their administration or release duration, especially if attempting to avoid frequent surgical interventions and interactions with the injury site. A way to resolve this may be by loading hydrogels with growth factors to support OEC functions related to regrowing axons [175]. Similarly, for growth factors that have a positive effect on OECs alone, and not the injury site, they could be used to treat OECs in vitro prior to in vivo transplantation (Figure 4). Importantly, hydrogels can, in some ways, act as reservoirs for growth factors so that they may be released over time via a concentration gradient, or with gradual degradation of the hydrogel.

Hydrogels are soft gels of polymeric substances that can hold significant amounts of water or aqueous media and therefore are easy to prepare with custom mechanical properties or “softness”. Importantly, the aqueous component of hydrogels presents an opportunity to load them with one or more soluble therapeutic agents, such as drugs or other bioactive molecules like growth factors, that are primed to deliver their payload at a sustained rate over time to an injury site. These hyaluronic acid hydrogels have been placed in vivo via transplantation in rats [176], injection in mice [177], or directly onto the injury site in mice [175]. Hydrogels loaded with nerve growth factor have also been used in rats to facilitate release of a growth factor gradient to accelerate axonal growth for peripheral nerve regeneration in vivo [175]. Thus, growth factors loaded onto hydrogels is a practical option to deliver them in a more targeted manner and to generate a growth factor concentration gradient to guide cells. The biodegradable nature of certain hydrogels also creates an opportunity to customise their degradation rate, thereby loading them with growth factors that will be delivered over a desired period of time in a slow or sustained release.

Use of osmotic pumps may be another delivery agent to supply OECs with exogenous growth factors after cell transplantation. This approach has been successfully used in rats with SCI to reduce secondary degeneration by delivering a sustained dose of VEGF + PDGF into the injury site [148].

Alternatively, nanoparticle-based growth factor delivery could be an option. Examples of nanoparticles include polymeric nanoparticles and liposomes [178], which are spherical vesicles carrying drugs and growth factors, to avoid degradation during transport and allow their accumulation at a target site [179,180,181]. In vitro studies show growth factor-loaded liposomes provide controlled release of growth factors over time, resulting in a concentration gradient [182,183]. Several researchers have shown that growth factors can be delivered into different injury sites using liposomes [180,184,185]. Liposomes loaded with FGF2 have been shown in contusion SCI rat models to penetrate the blood–spinal cord barrier, and facilitate spinal cord repair, resulting in motor function recovery [180]. Therefore, growth factor-loaded liposomes may be another practical option to deliver growth factors into a lesion site and/or provide a concentration gradient to stimulate cells.

## 4. Conclusions

There is an urgent need to generate therapies for treating SCI due to the number of individuals impacted and the severity of the injury impacts. OEC transplantation is one potential approach, but low cell survival and activity after integration may be limiting efficacy. Exploiting growth factors to stimulate OECs is proposed here as an effective method to improve therapeutic use of OECs; however, the challenge is to identify which growth factor(s) are most effective and how they can be applied during transplantation. A combination of scaffolds to physically support OECs and growth factors, and identification of most impactful growth factors, may lead to enhanced survival, integration, and efficacy of OECs in repairing spinal cord injury. Despite numerous studies supporting the promising application of growth factors in cellular stimulation and modulation, there is a clear need to focus research on exploring potential combinations and timing to maximize efficacy and optimize enhancement of OECs’ therapeutic potential.

## Figures and Tables

**Table 1:** Overview of the role, regulation, and target cells of different growth factors in SCI.

Growth Factor	Pre-Injury	Post-Injury	Secreted by	Target Cells	Biological Function
BDNF [154,155,156]	Low	Elevated	Astrocytes, neurons, macrophage, microglia	Neurons	Axonal growth, synaptic plasticity
TGF-β1 [157,158,159]	Present	Elevated	Microglia, macrophages	Astrocytes, microglia, macrophages	Glia scar formation, neuronal survival
FGF2 [160,161,162]	Low	Elevated	Astrocytes, neurons	Reactive astrocytes, astrocytes, neurons	Proliferation, differentiation, neurite outgrowth
PDGF [57,163,164,165]	Present	Elevated	Neurons, glial cells	Neurons, glia cells	Preventing neuronal apoptosis, reduced astrocyte proliferation, reduced permeability of blood–spinal cord barrier
VEGF [157,166,167]	Present	Low to elevated	Neurons, glia cells	Endothelial cells, neurons	Vascular permeability, neuroprotective, angiogenesis
GDNF [152,166,168,169,170]	Low	Elevated	Astrocytes	Neurons, macrophage, microglia	Axonal regeneration, Remyelination, neuroprotection
NGF [168,171,172,173]	Low	Elevated	Schwann cells, neurons, glial cells	Neurons	Neuroprotection
NT-3 [174]	Low	Unknown	Unknown	Oligodendrocyte, neurons	Proliferation, survival

## Data Availability

No new data were created or analysed in this study. Data sharing is not applicable to this article.
